# Associations of medical conditions, lifestyle and unintentional weight loss in early old age: The 1946 British Birth Cohort

**DOI:** 10.1371/journal.pone.0211952

**Published:** 2019-04-09

**Authors:** Yixiao Cao, Rebecca Hardy, Wahyu Wulaningsih

**Affiliations:** 1 MRC Unit for Lifelong Health and Ageing at UCL, London, United Kingdom; 2 Institute of Genetics and Molecular Medicine, University of Edinburgh, Edinburgh, United Kingdom; International University of Health and Welfare, School of Medicine, JAPAN

## Abstract

**Background:**

Unintentional weight loss in older people has been linked to increased risk of mortality. We aimed to investigate common medical conditions and lifestyle factors, including body fat distribution, as potential determinants of recent and prospective unintentional weight loss in early old age.

**Methods:**

From the Medical Research Council (MRC) National Survey of Health and Development (NSHD), we included a total of 2234 study members aged 60–64 with information on unintentional weight loss in 2006–2010. Of these, 2136 also had information on unintentional weight loss recorded in 2015. Logistic regression was conducted to examine the associations between medical conditions, lifestyle, and body fat distribution at age 60–64 and unintentional weight loss at age 60–64 and 69.

**Results:**

A total of 109 of 2234 study members had unintentional weight loss at ages 60–64, and 166 of 2136 at age 69. Never smoking was associated with lower risk of unintentional weight loss at age 60–64 (OR = 0.29, 95%CI = 0.12–0.68 compared to current smokers), and this association remained when adjusted for other determinants. Greater waist-hip ratio (OR = 0.95, 95%CI = 0.91–0.99) and body fat-lean mass ratio (OR = 0.96, 95%CI = 0.94–0.99) were associated with less likelihood of unintentional weight loss at age 60–64. Never smoking and greater hip circumference at age 60–64 were associated with lower odds of unintentional weight loss at age 69.

**Conclusions:**

Smoking status and body fat distribution may help identify those at risk of unintentional weight loss in early old age. Their benefit in interventions to prevent age-associated weight loss needs to be further investigated.

## Introduction

Unintentional weight loss is a common phenomenon occurring in 15–20% of older adults aged 65 and above[1, 2]. Some studies reported that excess unintentional weight loss was associated with adverse clinical consequences such as functional decline, infections, and decubitus ulcers [1, 3]. Rapid unintentional weight loss may also be an indication of advanced progression of illness [4]. Accumulating evidence shows unintentional weight loss to be associated with increased risk of all-cause mortality[2, 5–7], compared with intentional weight loss or weight stability[5].

There is no unanimous definition of unintentional weight loss. Unintentional weight loss is, however, generally accepted as an involuntary decline in total body weight, emphasising a condition without any clear reason, and excluding the expected weight loss as a result of treatment or illness[3, 5]. Some studies defined problematic weight loss as a 5% loss in actual body weight in 30 days and a 10% loss in 6 months[8, 9], whereas Gaddey & Holder (2014) defined it as more than a 5% reduction in body weight within 6 to 12 months[1]. Clay (2001) found that residents in nursing facilities exceeding a 5 lb unintentional weight loss should be considered unhealthy[10]. However, an involuntary 10 lb weight loss in the past year is part of the Freid index for frailty which has been linked with mortality[11], and which has, therefore, been used in many studies in older people.

Medical conditions such as cardiovascular disease (CVD), diabetes and cancer may precede unintentional weight loss at old ages[5, 7]. For example, Wannamethee et al. (2005) prospectively studied 7735 British men aged 56 to 75 years[7] and showed that 38% patients with CVD reported unintentional weight loss whereas 26% did not experience weight change. This study also showed that 10% of those with cancer and 11% with diabetes experienced unintentional weight loss[5]. A similar study estimated that cancer results in unintentional weight loss in at least 5% of patients[12]. However, observational studies have shown that in as many as 25% of cases of unintentional weight loss, no identifiable cause is found despite extensive investigations[2, 9].

The role of modifiable lifestyle factors in unintentional weight loss is less clear. Physical activity has been associated with intentional weight loss[7, 13], for example, walking with an energy expenditure of 2500kcal/week was an effective intervention to promote weight loss [13], but it is unknown how physical activity is associated with unintentional weight loss. Similarly, smoking cessation has been documented in research to be inversely associated with weight gain[14], and smokers have been found to weigh less than non-smokers on average[15] and more likely to experience unintentional weight loss than weight gain[7]. In one study, compared with individuals who experienced intentional weight loss, those who lost weight unintentionally were more likely to be of lower social class, be current smokers, and less physically activie and have higher rates of cancer and respiratory diseases[16].

Almost all studies investigating unintentional weight loss focused on those older than 65 years. Understanding the determinants of the phenomenon in early old age prior to age 65 may provide insight into potential prevention measures. Therefore, we used data from the Medical Research Council (MRC) National Survey of Health and Development (NSHD), which provides information about developmental and environmental influences from birth through to early old age, and enabled us to investigate the extent of unintentional weight loss in early old age and its determinants, particularly medical conditions and lifestyle factors as well as body fat distribution.

## Materials and methods

### Study sample

The Medical Research Council (MRC) National Survey of Health and Development (NSHD) is a socially stratified sample of 5326 singleton births delivered during one week in March 1946 in mainland Britain, with regular follow-up across life[17]. Between 2006 and 2010 (at ages 60–64), study members still alive and with a known current address in England, Scotland, or Wales were invited for an assessment at one of six clinical research facilities (CRF) or to be visited by a research nurse at home. Invitations were not sent to those who had died (N = 778), who were living abroad (N = 570), had previously withdrawn from the study (N = 594), or had been lost to follow-up (N = 564). Of the 2856 invited participants, 2234 was assessed with question of unintentional weight loss. In 2014–2015 (at ages 68–69 years), 957 study members had already dead, 620 had previously withdrawn permanently, 574 lived abroad, and 395 had remained untraceable. A total of 2148 participated in a home visit carried out by trained research nurses in 2015[18]. Ethical approval was obtained from the Greater Manchester Local Research Ethics Committee and the Scotland Research Ethics Committee. Written informed consent was obtained from the study member for each component of each data collection.

### Measurements

#### Unintentional weight loss

Unintentional weight loss was assessed by questionnaires administered by research nurses. At ages 60–64, study members were asked whether they had unintentionally lost more than 10 pounds of weight in the last year. This question was part of the Fried criteria for frailty[19], which has been linked to morbidity and mortality in older adults. At the home visits at age 69, multiple questions about weight change in the last year were asked (Figure A in S1 File). Two outcome variables were derived: unintentional weight loss of any amount, and unintentional weight loss of more than 10 pounds in the past year. Due to the small number of study members at age 69 who reported losing more than 10 pounds in weight (the same definition as the unintentional weight loss at age 60–64), we used any amount of unintentional weight loss in the past year as the outcome for age 69. A total of 2234 participants at age 60–64 and 2136 at age 69 years with complete information on weight loss were included in analyses (Fig A in S1 File).

#### Medical and lifestyle factors

At ages 60–64, study members reported any diagnosis of cancer, heart disease, and diabetes through self-report questionnaires. Total cholesterol, triglycerides and HbA1_C_ were measured in an overnight fasting 50-ml blood sample collected at the same age[17]. We dichotomised levels of total cholesterol (≤/>5.2 mmol/L) and triglycerides (≤/>2.26 mmol/L) based on the National Cholesterol Education Program guideline[20] and HbA1_C_ (≤/>48 mmol/mol) based on the cut-off for diabetes[21]. Cigarette smoking status (current smoker, ex-smoker, never smoker) and the frequency of leisure time physical activity were self-reported at age 60–64. For physical activity, participants reported the number of occasions on which they participated in sports, vigorous leisure activities and exercises over the past 4 weeks and we categorised physical activity into none, 1–4, and ≥ 5 times/month[22].

#### Body fat distribution

Waist and hip circumferences were measured at ages 60–64 and waist-hip ratio was defined as (waist circumference (cm)/ hip circumference (cm)×100%. Body composition was measured using a QDR 4500 Discovery DXA scanner (Hologic Inc, Bedford, MA, USA) at age 60–64 in the CRF; to optimise precision, scans were reviewed and centrally analysed (in Manchester) by a single operator (JA) using APEX 3.1 software (Hologic Inc., Bedford, MA, USA)[23]. Measures of fat (whole body, android and gynoid) and lean mass (whole body and appendicular) were obtained and converted into kilograms. Fat/lean ratio was derived and multiplied by 100.

#### Other covariates

Childhood socioeconomic position was measured by father’s occupational social class when study members were aged 4, and adult social class by participants own occupation in 1999 (at age 53 years). Data was categorized based on the Registrar General’s classification[23] and for this study classes I to III non-manual were grouped as non-manual social class and classes III manual to V to manual.

### Statistical analyses

Logistic regression models were used to examine the associations of medical and life style factors and adiposity with unintentional weight loss at ages 60–64 years (more than 10 pounds) and age 69 years (any amount). In the initial analysis, no evidence of any interactions by sex were found, and therefore all results are presented for the overall study population while including sex in the model as an adjustment variable. Therefore, models for each potential determinant were adjusted for sex (model 1), and additionally for childhood and adulthood socioeconomic position (model 2). A multivariable model was then fitted including all medical conditions and life style factors in the same model with each body fat distribution measure included separately (model 3) because of the high correlation (up to 80%) between these variables. Complete case analysis was conducted.

Because lifestyle may be influenced by diagnosis of chronic diseases, we repeated our analysis for lifestyle factors while excluding those diagnosed with heart disease, diabetes and cancer.

When results from multivariable analyses differed from univariable analyses, additional sensitivity analyses were performed repeating the analyses for a given predictor while adjusting for one covariate at a time, to examine which factor most strongly influenced the association between predictor and unintentional weight loss.

## Results

Characteristics of study participants at age 60–64 by unintentional weight loss status at age 60–64 are presented in Table 1. Of 2234 participants aged 60–64 years, 109 (4.88%) participants reported unintentional weight loss of more than 10 pounds, whereas 166 (7.77%) of 2136 participants at age 69 years reported unintentional weight loss but only 59 of these 166 (35.54%) lost more than 10 pounds. There were greater proportions of current smokers and those who were physically inactive in the unintentional weight loss group compared with the group without weight loss, whereas the proportion of ex-smoker was higher in the latter group (Table 1).

**Table 1:** Characteristics of the samples with unintentional weight loss medical conditions at and life style factors at age 60–64.

	Unintentional weight loss at age 60–64				Total (N = 2234)	
	Yes (age 60–64) (N = 109)		No (age 60–64) (N = 2125)			
	N total	Mean(S.D.)/ N (%)	N total	Mean (S.D.)/ N (%)	N total	Mean (S.D.)
**Waist circumference, cm**	107	94.52(16.29)	2104	96.57(12.67)	2211	96.47(12.87)
**Hip circumference, cm**	108	104.47(12.24)	2101	105.82(9.64)	2209	105.76(9.78)
**Waist-hip ratio**	107	0.90(0.09)	2101	0.91(0.08)	2208	0.91(0.08)
**Fat, kg**	72	11.87(29.13)	1581	8.26(22.69)	1658	8.46(23.01)
**Lean, kg**	65	44.50(10.77)	1490	45.16(10.29)	1555	45.13(10.30)
**Fat-lean ratio**	65	0.56(0.23)	1490	0.62(0.23)	1555	0.61(0.23)
**Diabetes**	109	6(5.5%)	2125	136(6.4%)	2234	142(6.36%)
**Cancer**	109	11(10.09%)	2125	167(7.86%)	2234	178(7.97%)
**Heart disease**	92	6(6.52%)	1873	120(6.41%)	1965	126(6.41%)
**Total cholesterol (mmol/L)**	95	5.52(1.11)	1962	5.67(1.20)	2057	5.67(1.19)
**Triglyceride (mmol/L)**	91	1.31(0.76)	1883	1.33(0.78)	1974	1.33(0.78)
**HbA1c (mmol/mol)**	99	5.93(0.79)	1938	5.84(0.71)	2037	5.85(0.71)
**Physical activity**	103		2066		2169	
** None**		68(66.02%)		1317(63.71%)		1385(63.86%)
** 1–4 times/month**		10(9.71%)		287(13.89%)		297(13.69%)
** 5 or more times/month**		25(24.27%)		462(22.36%)		487(22.45%)
**Cigarette smoking status**	97		1937		2034	
** Current smoker**		21(21.65%)		208(10.74%)		229(11.26%)
** Ex-smoker**		52(53.61%)		1096(56.58%)		1148(56.44%)
** Lifelong Non-smoker**		24(24.74%)		633(32.68%)		657(32.30%)
**Childhood social class**	100		2022		2122	
** Non- manual**		47(47%)		937(46.34%)		984(46.37%)
** Manual**		53(53%)		1085(53.66%)		1138(53.63%)
**Adult social class**	108		2115		2223	
** Non-manual**		74(68.52%)		1485(70.21%)		1559(70.13%)
** Manual**		34(31.48%)		630(29.79%)		664(29.87%)

### Unintentional weight loss at ages 60–64

Table 2 displays odds ratios for unintentional weight loss at age 60–64 for each of the medical and life style factors and body fat distribution. No association was found between current medical conditions and unintentional weight loss at age 60–64 in sex-adjusted models (model 1) or when further adjusted for childhood and adult social class (model 2). When including all medical conditions and lifestyle factors in model 3, higher total cholesterol was associated with a decreased odds of unintentional weight loss (OR = 0.77 per mmol/L, 95%CI = 0.60–0.98). When compared with current smokers, both ex-smokers and non-smokers were less likely to have unintentional weight loss (OR = 0.38, 95%CI = 0.21–0.69) (Table 2). These associations remained after adjustment for childhood and adulthood social class and were more pronounced with adjustment for all other factors.

**Table 2:** Odds ratio for unintentional weight loss at age 60–64 by medical conditions and life style factors and central adiposity at 60–64 years.

	Model 1^a^		Model 2^b^		Model 3 ^c^	
	N	OR(95%CI)	N	OR(95%CI)	N	OR (95%CI)
**Diabetes**	2234	0.85(0.37–1.98)	2113	0.95(0.41–2.21)	1525	0.68(0.21–2.19)
**Cancer**	2234	1.31(0.69–2.50)	2113	0.89(0.40–1.95)	1525	1.50(0.66–3.41)
**Heart disease**	1965	0.98(0.42–2.29)	1861	0.91(0.36–2.30)	1525	0.32(0.07–1.39)
**Total Cholesterol (mmol/L)**	2057	0.89(0.74–1.07)	1945	0.88(0.73–1.07)	1525	0.77(0.60–0.98)
**Triglyceride(mmol/L)**	1974	0.97(0.74–1.29)	1866	0.94(0.69–1.27)	1525	0.87(0.58–1.30)
**HbA1c(mmol/mol)**	2037	1.15(0.91–1.46)	1925	1.16(0.91–1.48)	1525	1.18(0.85–1.64)
**Physical activity**	2169		2051		1525	
** None**	1385	1.0 reference	1308	1.0 reference	955	1.0 reference
** 1–4 times/month**	297	0.67(0.34–1.33)	280	0.58(0.27–1.24)	219	0.93(0.42–2.06)
** 5 or more times/month**	487	1.04(0.65–1.68)	463	1.03(0.63–1.71)	351	1.26(0.69–2.32)
**Cigarette smoking status**	2034		1925		1525	
** Current smoker**	229	1.0 reference	218	1.0 reference	175	1.0 reference
** Ex-smoker**	1148	0.47(0.28–0.80)	1092	0.40(0.23–0.71)	863	0.58(0.28–1.17)
** Non-smoker**	657	0.38(0.21–0.69)	615	0.35(0.18–0.67)	487	0.29(0.12–0.68)
**Adiposity**						
** Waist circumference, cm**	2211	0.99(0.97–1.00)	2091	0.99(0.97–1.01)	1521	0.96(0.94–0.99)
** Hip circumference, cm**	2209	0.98(0.96–1.00)	2089	0.99(0.97–1.01)	1519	0.97(0.94–1.00)
** Waist-hip ratio, %**	2208	0.98(0.95–1.01)	2088	0.98(0.95–1.01)	1518	0.95(0.91–0.99)
** Fat mass, kg**	1653	1.01(0.99–1.01)	1557	1.01(0.99–1.01)	1192	1.01(0.99–1.02)
** Lean mass, kg**	1555	0.98(0.94–1.02)	1467	0.99(0.95–1.03)	1122	0.92(0.87–0.98)
** Fat-lean ratio, %**	1555	0.97(0.96–0.99)	1467	0.98(0.96–0.99)	1122	0.96(0.94–0.99)

^a^ Adjusted for sex

^b^ Adjusted for sex, childhood and adulthood socio-economic status

^c^ Adjusted for sex, childhood and adulthood socio-economic status, cancer, HbA1c, heart disease, cholesterol, triglyceride, cigarette smoking status, physical activities, unless the variable are used as the predictor.

* P-value<0.05

In the sensitivity analysis excluding those diagnosed with heart disease, diabetes or cancer, the association with smoking status remained significant (OR = 0.33, 95% CI = 0.13–0.82 for never compared to current smokers) but that with total cholesterol did not (data not shown). Correspondingly, when including one covariate at a time in the model with cholesterol as the main predictor, heart disease was the factor which strengthened the association between cholesterol and unintentional weight loss. When adjusted for heart disease, every one-unit increase in total cholesterol resulted in 20% decreased odds of unintentional weight loss (95%CI = 0.65–0.99) (Table A in S1 File).

With regards to body fat distribution, higher waist and hip circumferences were associated with lower odds of unintentional weight loss at age 60–64 in model 3. Additionally, a 1% increase in waist-hip ratio incurred a decreased odds of unintentional weight loss by 5% when controlling for medical and lifestyle factors (OR = 0.95, 95%CI = 0.94–0.99). Lean mass showed similar results in the multivariable model with a one unit increase in lean mass associated with an 8% decreased odds of unintentional weight loss (OR = 0.92, 95%CI = 0.87–0.98). Similarly, higher fat-lean ratio was associated with reduced odds of unintentional weight loss in the sex-adjusted model and this association was hardly affected by further adjustments (Table 2).

### Unintentional weight loss at age 69

No strong evidence of an association of medical conditions at ages 60–64 years with unintentional weight loss at age 69 years was observed (Table 3). Lower odds of unintentional weight loss were found for those reporting participations in physical activity 5 or more times/month in model 1 and model 2, compared with those reporting no physical activity (OR = 0.55, 95%CI = 0.34–0.88; OR = 0.56, 95%CI = 0.34–0.92 in model 1 and 2, respectively). This association was slightly attenuated in model 3 (OR = 0.57, 95%CI = 0.32–1.01) and this was mainly due to smoking status (Table B in S1 File). Low odds of unintentional weight loss at age 69 were observed in all models for both ex-smoker and non-smoker groups (p<0.05) compared with smokers (OR = 0.36, 95%CI = 0.19–0.70 for never compared to current smokers), and non-smokers tended to have a lower risk than ex-smokers (Table 3). When we excluded those diagnosed with heart disease, diabetes or cancer, an association remained for physical activity (OR = 0.52, 95% CI = 0.27–0.99 for 5 or more times/month compared to none) and smoking status (OR = 0.38, 95% CI = 0.19–0.76 for never compared to current smokers) and no association was observed for other factors (data not shown).

**Table 3:** Odds ratio for unintentional weight loss at age 69 by medical conditions and life-style factors and central adiposity at 60–64 years.

	Model 1^a^		Model 2^b^		Model 3 ^c^	
	N	OR(95%CI)	N	OR(95%CI)	N	OR(95%CI)
**Diabetes**	2136	0.70(0.32–1.54)	2013	0.75(0.34–1.65)	1317	0.69(0.21–2.25)
**Cancer**	2136	0.77(0.40–1.50)	2013	0.85(0.44–1.66)	1317	1.11(0.52–2.38)
**Heart disease**	1789	1.52(0.77–3.03)	1692	1.70(0.85–3.40)	1317	1.64(0.72–3.74)
**Total Cholesterol (mmol/L)**	1746	0.99(0.84–1.15)	1652	0.99(0.84–1.16)	1317	1.03(0.85–1.25)
**Triglyceride (mmol/L)**	1686	0.89(0.69–1.15)	1595	0.85(0.64–1.11)	1317	0.76(0.54–1.08)
**HbA1c (mmol/mol)**	1720	0.98(0.75–1.28)	1626	0.99(0.75–1.30)	1317	0.98(0.65–1.49)
**Physical activity**	1828		1728		1317	
** None**	1121	1.0 reference	1058	1.0 reference	790	1.0 reference
** 1–4 times/month**	271	0.78(0.47–1.29)	255	0.79(0.46–1.34)	203	0.81(0.44–1.48)
** 5 or more times/month**	436	0.55(0.34–0.88)	415	0.56(0.34–0.92)	324	0.57(0.32–1.01)
**Cigarette smoking status**	1842		1739		1317	
** Current smoker**	191	1.0 reference	178	1.0 reference	134	1.0 reference
** Ex-smoker**	1050	0.44(0.27–0.71)	997	0.44(0.27–0.73)	758	0.42(0.23–0.75)
** Non-smoker**	601	0.39(0.23–0.66)	564	0.36(0.21–0.64)	425	0.36(0.19–0.70)
**Adiposity**						
** Waist circumference, cm**	1864	0.98(0.97–0.99)	1762	0.98(0.97–0.99)	1312	0.99(0.97–1.01)
** Hip circumference, cm**	1863	0.97(0.95–0.99)	1761	0.97(0.95–0.99)	1311	0.97(0.95–0.99)
** Waist-hip ratio, %**	1862	1.00(0.98–1.03)	1760	1.01(0.98–1.03)	1310	1.01(0.98–1.03)
** Fat, kg**	1463	1.00(0.99–1.01)	1380	1.00(0.99–1.01)	1069	1.00(0.99–1.01)
** Lean, kg**	1379	0.96 (0.93–0.99)	1304	0.97(0.93–1.00)	1008	0.98(0.94–1.03)
** Fat-lean ratio, %**	1379	0.99(0.97–1.00)	1304	0.99(0.98–1.00)	1008	0.99(0.97–1.01)

^a^ Adjusted for sex

^b^ Adjusted for sex, childhood and adulthood socio-economic status

^c^ Adjusted for sex, childhood and adulthood socio-economic status, cancer, HbA1c, heart disease, cholesterol, triglyceride, cigarette smoking status, physical activities, unless the variable are used as the predictor.

* P-value<0.05

In terms of body fat distribution and composition at ages 60–64, associations with unintentional weight loss at age 69 years were weak. Higher waist circumference was associated with lower odds of weight loss in model 1 and model 2 (OR = 0.98, 95%CI = 0.97–0.99 in two models), but no longer associated with unintentional weight loss when adjusted for other covariates. Hip circumference at age 60–64 but not waist-hip ratio was associated with unintentional weight loss, despite a slight decrease in the estimate compared with age 60–64 (OR = 0.97, 95%CI = 0.95–0.99). Additionally, lean mass was associated with unintentional weight loss in the sex-adjusted model (OR = 0.96, 95%CI = 0.93 = 0.99), but not in other two models, and it was likely that the attenuation was due to as adjustment for heart disease, physical activity, and smoking status (Table C in S1 File).

## Discussion

In the NSHD, a British birth cohort study, we found evidence that smoking in early old age was associated with unintentional weight loss in cross-sectional and prospective analyses. The associations were not iattenuated by medical status and other potential confounders. Both higher levels of physical activity (5 times and above per month) and greater hip circumference were associated with reduced risk of unintentional weight loss in later life.

Age is a key determinant of unintentional weight loss in older people. The British Regional Heart Study assessed 4713 older British men from 1992 to 1996 and demonstrated that with increasing age, there was an increase in those reporting unintentional weight loss[16]. Diminishing deposition of adipose tissue may play a role in this phenomena, but a gradual loss of muscle tissue associated with aging together with a decrease in physical activity may also contribute to weight loss[16]. Consistent with this, our study found that increasing hip circumference at age 60–64 was associated with reduced likelihood of unintentional weight loss at age 69. Our study also found that at ages 60–64, body fat distribution was cross-sectionally strongly associated with unintentional weight loss. As lean body mass consists of bones, ligaments, organs and muscles, increases in lean mass may result from increases in each of these components, any of which may be linked with the decreasing likelihood of unintentional weight loss.

Previous studies demonstrated that lower socioeconomic position was related to unintentional weight loss[1, 5]. It is generally accepted that lower socioeconomic position could predispose to increased risk of medical condition and less likelihood of receiving early diagnosis and treatment, thus aggravating the effects of poor health including weight loss. However, in this study, neither childhood nor adult social class had a direct relationship with unintentional weight loss.

Our study corroborated the notion that cigarette smoking is associated with increased risk of unintentional weight loss. This is consistent with a cross-sectional study which assessed 300 older adults in the Netherlands and demonstrated that smokers were more likely to experience weight loss compared with those who never smoked, and that smoking was associated with undernutrition (OR = 2.56)[14]. The Lung Health Study conducted in 5,887 smokers aged 35–60 from 1986 to 1994 across the United States, reported major weight gain to be strongly related to smoking cessation, with an estimated 33% of sustained quitters gaining >10 1bs over 5 years[24]. The mechanism by which cigarette smoking influences weight loss has well documented and is attributed to both decreased caloric intake and accelerated energy expenditure[14, 25]. It is generally accepted that consuming nicotine results in the reduction of taste perception and appetite[14] and increases the feeling of fullness[26]. When quitting smoking, the metabolic effects of nicotine abstinence could boost appetite and prevent the occurrence of unintentional weight loss in old age. Additionally, smokers may have undetected subclinical disease which may have explained the weight loss.

In contrast to studies which have demonstrated that greater levels of physical activity promote weight loss[14, 27], our research indicated that greater levels of physical activity were prospectively associated with reduced risk of unintentional weight loss. This suggests that the influence of physical activities on weight loss differs depending on weight loss intention; greater physical activity will result in weight loss if an individual is intentionally losing weight, but may be protective against losing weight unintentionally. This may be explained by the preventive role of physical activity against muscle loss, which contributed to total body weight. However, the effect of physical activity on prospective weight loss in our analysis weakened when adjusted by smoking. Since individuals who participate in greater levels of physical activity often adopt other healthy lifestyle, e.g. abstinence from smoking, this finding may suggest that the association between physical activity and unintentional weight loss in early old age may be confounded by smoking, or otherwise reflect the strong correlation between a lack of physical activity and smoking. Although further studies are needed to confirm any causal association, linking these risk behaviours to unintentional weight loss may contribute to advocating healthy life styles and identifying points of intervention. For example, given the similar associations of smoking and physical activity with unintentional weight loss, it may be more beneficial to use multiple risk factors to identify the subgroup of individuals at highest risk which may benefit from preventative measures and more frequent monitoring.

The major strength of this study is that NSHD is a prospective cohort with information allowing investigation of medical conditions and life styles with unintentional weight loss cross-sectionally at age 60–64 and also prospectively at age 69. Information on potential confounders was available in this study.

The NSHD sample is representative of the general British-born population, and therefore our findings are likely relevant to other high-income countries[28].However, as we did not have weight measurement annually, our assessment of unintentional weight loss was dependent on self-report and we were unable to use other definitions of unintentional weight loss[1, 29]. Use of self-report may be subject to recall bias, however, previous research showed self-reports on weight change intention to be fairly reliable and accurate[16]. In addition, the number of those with unintentional weight loss was limited, especially at age 69, and we were thus unable to use the same cut-off for unintentional weight loss at age 69 as at age 60–64. We therefore cannot directly compare results at the two ages. In addition, the comparison group comprised those without unintentional weight loss and may therefore include those undergoing intentional weight loss, although the numbers were small at age 69 (Fig A in S1 File). We used information on self-reported medical conditions and may therefore have missed subclinical disease which may affect weight. Additionally, our study only demonstrated association and not causation and requires further mechanistic or causal studies to clarify any potential target for intervention to maintain healthy weight in older ages.

## Conclusion

We identified some lifestyle and body composition determinants of unintentional weight loss in early old age in a British sample which may guide future research on the development of effective interventions which can help prevent problematic weight loss among older people and reduce the associated costs. However, more work is needed to understand the mechanisms of unintentional weight loss through incorporating other risk factors and using a unified definition of unintentional weight loss.

## Supporting information

S1 File Fig A. Overview of weight change assessed at age 69. Table A. Odds ratio for unintentional weight loss at age 60–64 by total cholesterol at 60–64 years, adjusted for each variable in the left-hand column separately. Table B. Odds ratio for unintentional weight loss at age 69 by physical activity 5 or more times/month at 60–64 years, adjusted for each variable in the left-hand column separately. Table C.Odds ratio for unintentional weight loss at age 69 by lean mass at 60–64 years, adjusted for each variable in the left-hand column separately.
(DOCX)
