# Supplementary material for: Associations of medical conditions, lifestyle and unintentional weight loss in early old age: The 1946 British Birth Cohort
Source: PLoS One. 2019 Apr 9;14(4):e0211952. doi: 10.1371/journal.pone.0211952 (PMC6456161; doi:10.1371/journal.pone.0211952)
Supplement: S1 File — Fig A. Overview of weight change assessed at age 69. Table A. Odds ratio for unintentional weight loss at age 60–64 by total cholesterol at 60–64 years, adjusted for each variable in the left-hand column separately. Table B. Odds ratio for unintentional weight loss at age 69 by physical activity 5 or more times/month at 60–64 years, adjusted for each variable in the left-hand column separately. Table C.Odds ratio for unintentional weight loss at age 69 by lean mass at 60–64 years, adjusted for each variable in the left-hand column separately. (DOCX) [file pone.0211952.s001.docx]

**S1 File.**

**Fig A. Overview of weight change assessed at age 69.**

5362 live births recruited in 1946

2136 assessed weight change at 2015 (aged 69 years)

418 had weight loss

577 increased weight

1141 not changed weight

252 lost weight intentionally

166 had unintentional weight loss

47 decreased by <5 lbs

59 decreased by >10 lbs

60 decreased by 5-10 lbs

**Table A. Odds ratio for unintentional weight loss at age 60-64 by total cholesterol at 60-64 years, adjusted for each variable in the left-hand column separately.**

| **Total Cholesterol** | **OR (95%CI)** | **p-value** |
| --- | --- | --- |
| Diabetes | 0.87(0.71-1.07) | 0.176 |
| HbA1c | 0.90(0.74-1.10) | 0.314 |
| Cancer | 0.87(0.72-1.07) | 0.186 |
| Heart disease | 0.80(0.65-0.99) | 0.049* |
| Triglyceride | 0.88(0.72-1.07) | 0.215 |
| Physical activities | 0.97(0.76-1.24) | 0.825 |
| Cigarette smoking status (current smoker) | 1.14(0.73-1.79) | 0.563 |

**Table B. Odds ratio for unintentional weight loss at age 69 by physical activity 5 or more times/month at 60-64 years, adjusted for each variable in the left-hand column separately.**

| **Physical activity**  **5 or more times/month** | **OR (95%CI)** | **p-value** |
| --- | --- | --- |
| Diabetes | 0.60(0.46-0.99) | 0.046* |
| HbA1c | 0.59(0.36-0.99) | 0.045* |
| Cancer | 0.52(0.31-0.88) | 0.016* |
| Heart disease | 0.49(0.28-0.85) | 0.012* |
| Triglyceride | 0.58(0.35-0.97) | 0.040* |
| Cholesterol | 0.58(0.35-0.97) | 0.036* |
| Cigarette smoking status (current smoker) | 0.80(0.16-3.85) | 0.776 |

**Table C.Odds ratio for unintentional weight loss at age 69 by lean mass at 60-64 years, adjusted for each variable in the left-hand column separately.**

| **Lean mass** | **OR (95%CI)** | **p-value** |
| --- | --- | --- |
| Diabetes | 0.97(0.93-1.00) | 0.074 |
| HbA1c | 0.98(0.94-1.01) | 0.207 |
| Cancer | 0.96(0.93-0.99) | 0.048* |
| Heart disease | 0.99(0.95-1.03) | 0.549 |
| Triglyceride | 0.96(0.93-1.00) | 0.065 |
| Cholesterol | 0.96(0.93-0.99) | 0.033* |
| Physical activities | 0.97(0.93-1.02) | 0.260 |
| Cigarette smoking status (current smoker) | 0.98(0.94-1.03) | 0.409 |
